# Changes in Rod and Frame Test Scores Recorded in Schoolchildren during Development – A Longitudinal Study

**DOI:** 10.1371/journal.pone.0065321

**Published:** 2013-05-27

**Authors:** Jeff Bagust, Sharon Docherty, Wayne Haynes, Richard Telford, Brice Isableu

**Affiliations:** 1 Visiting Researcher, School of Health and Social Care, Bournemouth University, Boumemouth, United Kingdom; 2 Anglo-European College of Chiropractic, Bournemouth, United Kingdom; 3 Canberra University, Canberra, Australia; 4 Australian National University, Canberra, Australia; 5 University of Paris-Sud. UR CIAMS EA 4532–Motor Control and Perception Team, Orsay, France; University of Lincoln, United Kingdom

## Abstract

The Rod and Frame Test has been used to assess the degree to which subjects rely on the visual frame of reference to perceive vertical (visual field dependence- independence perceptual style). Early investigations found children exhibited a wide range of alignment errors, which reduced as they matured. These studies used a mechanical Rod and Frame system, and presented only mean values of grouped data. The current study also considered changes in individual performance. Changes in rod alignment accuracy in 419 school children were measured using a computer-based Rod and Frame test. Each child was tested at school Grade 2 and retested in Grades 4 and 6. The results confirmed that children displayed a wide range of alignment errors, which decreased with age but did not reach the expected adult values. Although most children showed a decrease in frame dependency over the 4 years of the study, almost 20% had increased alignment errors suggesting that they were becoming more frame-dependent. Plots of individual variation (SD) against mean error allowed the sample to be divided into 4 groups; the majority with small errors and SDs; a group with small SDs, but alignments clustering around the frame angle of 18°; a group showing large errors in the opposite direction to the frame tilt; and a small number with large SDs whose alignment appeared to be random. The errors in the last 3 groups could largely be explained by alignment of the rod to different aspects of the frame. At corresponding ages females exhibited larger alignment errors than males although this did not reach statistical significance. This study confirms that children rely more heavily on the visual frame of reference for processing spatial orientation cues. Most become less frame-dependent as they mature, but there are considerable individual differences.

## Introduction

Postural stability (balance), orientation and the organisation of human movement require a complex synthesis of sensory signals from internal gravity, movement, muscle and joint receptors, and external cues, principally visual, to maintain balance. The choice of appropriate Frames of Reference (FoR) is crucial to reduce spatial and temporal uncertainty during the process of aligning coordinate systems of the body or part of it (egocentred ones) with exocentered FoRs. At the perceptual level the rod and frame test provides a measure of the influence of the visual FoR upon a subject's ability to determine vertical or upright. It allows the effect of the visual FoR on postural orientation (i.e., the extent to which subjects align their body axis on axes of the visual FoR) to be measured, which in turn influences the perceived orientation of the verticality of the rod. This test was developed in the 1950's by Witkin [1] as one of a battery of tests designed to classify cognitive styles. The ability of subjects to align a rod to vertical in the presence of a tilted surrounding frame, and their ability to identify hidden figures in a distracting background in the Embedded Figures Test was used to classify subjects as Field Dependent - strongly influenced by the visual environment, or Field Independent whose perception of vertical was less affected by the visual Frame of Reference [2].

Witkin et al. [3] showed that the influence of the surrounding frame upon the accuracy of judging vertical alignment using the Rod and Frame Test fell between the ages of 8 and 17 years, indicating lessening dependence on peripheral static visual cues of orientation (FoR). They also demonstrated gender differences with males exhibiting consistently lower errors than females [3]. These findings have been largely supported by subsequent investigations in children[4],[5],[6].

The construct of Field Dependence-Independence as a measure of cognitive style includes tests such as the Embedded Figures Test and the Articulation of Body Concept (ABC) [7] in addition to the Rod and Frame Test. However, a study in which a range of perceptual and cognitive tests were compared raised questions concerning the equivalence of the perceptual Rod and Frame Test and the cognitive Embedded Figures Test [8]. As a result, it has been suggested that the Rod and Frame Test is more of a measure of spatial ability than cognitive style [9],[10]. Furthermore, a longitudinal study of schoolchildren made at intervals between the ages of 3 and 18 involving the Rod and Frame Test, Embedded Figures Test, and personality tests, concluded that below the age of 7 the Rod and Frame Test is not a valid indicator of Field Dependence-Independence [9]. Since the current work was concerned solely with the Rod and Frame Test and the effects of a tilted surrounding frame on the perception of vertical, we have used the term Frame Dependent-Independent [5] to describe the results instead of Field Dependent-Independent.

Nyborg[11] noted that the development of the perception of vertical has received little attention since Witkin's [3] study which used group means or median scores to show trends in the sample population over time. The current investigation was carried out as part of the LOOK (Lifestyle Of Our Kids) project, a large longitudinal study following the development of a group of Australian schoolchildren between the ages of 7 and 12 years [12],[13]. The study incorporated a modern computer-based version of the Rod and Frame Test, and provided the opportunity to revisit Witkin's findings in greater detail and to follow the changes in performance of individual children during these formative years.

## Methods

### Subjects

The subjects for this study were participants in the Lifestyle of our Kids (LOOK) longitudinal study [12] attending elementary (primary) schools in an Australian education jurisdiction. The children were community dwelling boys and girls from suburbs where family income approximated the Australian average. Recordings were made from each child at 2 year intervals between 2005 and 2009 in Grade 2 (age 7–8 years), Grade 4 (9–10 years) and Grade 6 (11–12 years). There was a total of 419 subjects (214 male, 205 female) for which complete data sets were obtained.

The LOOK study was approved by the Australian Capital Territory Health and Community Care Human Research Ethics Committee (ref. ETH.9/05.697) as well as the Ethics Committee of the Australian Institute of Sport (ref. 20060606). Written parental and child consent was obtained for all measures in the study, and the children understood that their participation was entirely voluntary.

The children in the LOOK study were divided into two groups who followed different physical exercise programs at school. The LOOK study recorded many parameters including anthropometry, fitness and physical activity, as well literacy and numeracy. The Rod and Frame Test was included as one of the range of measurements.

The LOOK design included schools that varied in their physical education programs, some schools having specialist physical educators and others not. However, we have no reason to suspect that this had any influence on the outcomes of our study, as there were no differences in the course of development of RFT alignment outcomes over the four years of investigation. A general linear mixed methods model was applied to show that the school physical education program had no effect on the development of alignment outcomes over the four years of the study. There was no evidence of any difference in outcomes at baseline, nor in the change in RFT alignment outcomes between Grades 2 and 6 (p = 0.6) for difference between the programs (unpublished LOOK study report, the details of which are available on request to the corresponding author). The data from all the schools involved in the LOOK study have therefore been included in this analysis.

### Rod and Frame Test

The Computer Rod and Frame (CRAF) test used in these investigations was a modified version of the program described by Bagust et al. [14]. The program was written in Visual Basic, and ran on a portable laptop computer. The subject was presented with a square white frame on a plain black background. Within the frame a single white line could be rotated around its centre in either clockwise or counter clockwise directions by depressing the right or left mouse buttons respectively. The rod rotated in steps of 0.5° for each mouse key press.

The Rod and Frame display was presented to the subjects on a 17 inch flat screen monitor. A black cylinder 13 inches diameter and 18 inches long placed in front of the screen formed an optical tunnel. One aspect of the cylinder abutted the screen and the other opening allowed the subject to rest their chin inside the tube. A black velvet cloth covered the subject's head to eliminate any light, removing any peripheral visual cues of verticality. The frame subtended a visual angle of 25°.

The subject was seated with the screen and viewing cylinder at eye level. Children were instructed to look through the viewing tube and asked what they saw. They were then asked to imagine the rod was a rocket ship that had to be pointed straight up towards an imaginary moon. They were told if the rocket did not point straight up then it would miss the moon and crash. The child was given 2 practice sessions (one of each frame direction) to rotate the rocket ship (rod) before launch. Following the practice screens their response was reviewed and they were then shown the true vertical position of the “rocket ship” and how their alignment differed from it.

The test operator observed the practice presentations and any child showing signs of either not having understood the instructions, or aligning the rod to the frame, was further tutored. In this way, time was spent establishing a reasonable understanding of the task. Less graphic instructions were given in Grades 4 and 6 as children appeared to understand the verbal instructions more readily. The results of the practice presentations were discarded for the analysis.

The test itself consisted of a pre-programmed sequence of 10 rod and frame tasks presented in random order and consisting of 5 replicates each of the frame tilted 18° in a clockwise (+18°) and counter clockwise (−18°) direction from the vertical. The rod was initially positioned at an angle of either +20° or −20° from vertical. To eliminate possible bias and learning effects the order of display presentation was automatically selected by the computer at the beginning of each trial from a bank of 4 randomised sequences. When the child was satisfied that the rocket (rod) was pointing straight up (vertical) they pressed the space bar on the keyboard, clearing the screen for 1 second before the next rod and frame were displayed. At the same time the computer recorded the angle of the rod relative to vertical (0°) and the time taken to make the alignment judgement. Negative error values indicate a counter clockwise error and positive values a clockwise error.

The mean of the ten absolute (unsigned) errors for each subject were calculated. This provides a single value for each subject which combines the clockwise and counter clockwise frame effects and enables comparison with other published studies, but loses detail concerning the effects of the different directions of frame tilt. This was addressed by also calculating the mean signed (arithmetic or constant error [15]) alignment error from the ten test presentations for each subject, a measure which retains the directional information, but suffers from the risk of minimising the size of large symmetrical errors (eg the mean of +10° and −10° errors  = 0°). For each individual, additional calculations were made of the mean signed error and SD for the 5 replicates with the frame tilted clockwise (iMean^+^, iSD^+^) and counter clockwise (iMean^−^, iSD^−^). The iSD values provided a measure of the consistency with which the subject was targeting their response.

### Alignment strategies

**Figure 1:**
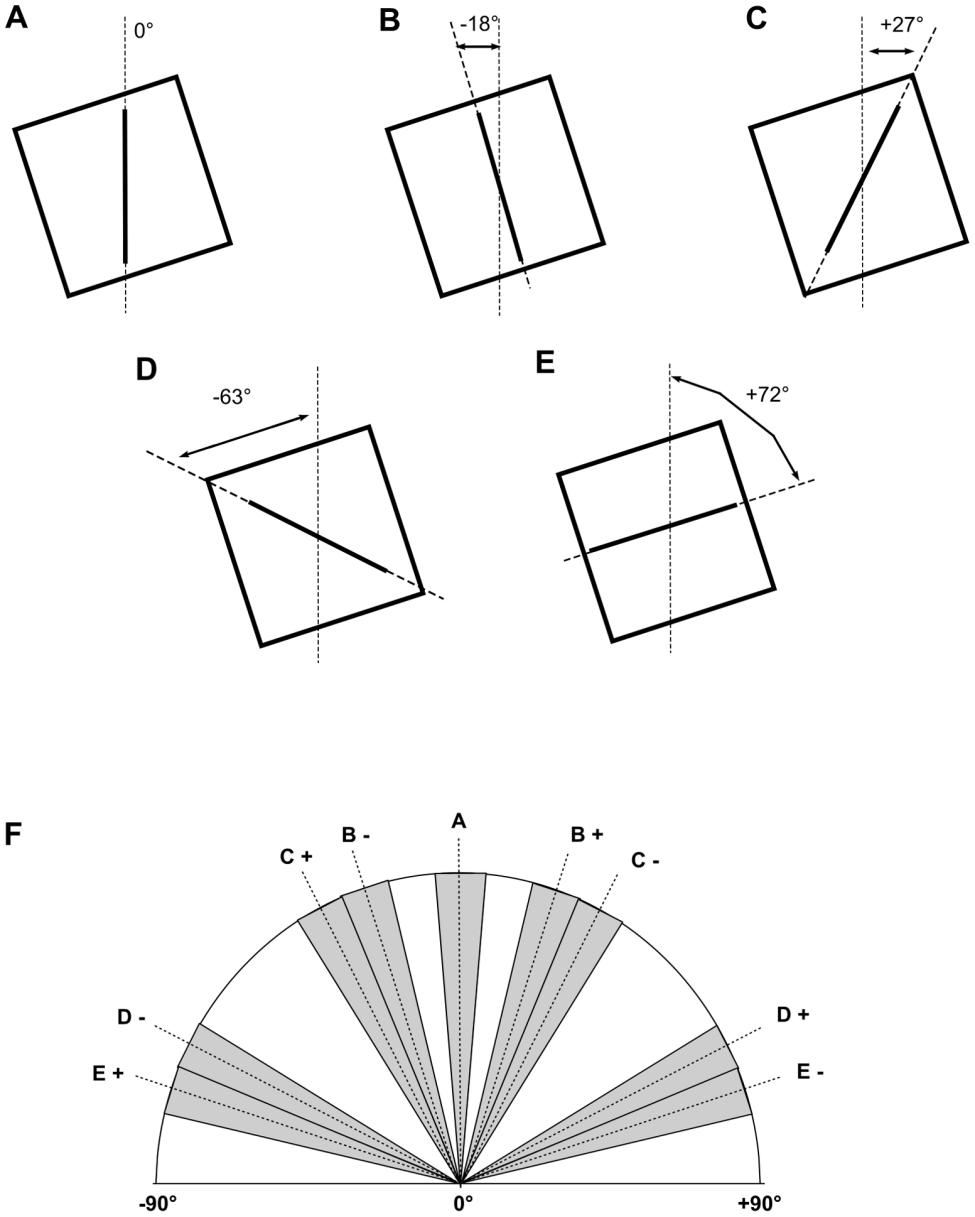
A–E. Frame alignment errors with frame tilt −18° (counter clockwise). Rod aligned to –A, vertical; B, lateral side; C, upper right corner; D, upper left corner; E, upper side. A mirror set of errors is generated with the frame tilt clockwise (+18°). F, shaded −9° sectors used to quantify the corresponding alignment strategies in A–E; unshaded–sectors classified as ‘other’. Sectors marked + frame tilt +18°; sectors marked – frame tilt −18°.

Alignment of the rod to non-vertical components of the frame produced characteristic errors that allowed the alignment strategy used for each individual line placement to be identified (Fig 1A–E). In addition to vertical (0°), four other possible targeting strategies were identified (alignment to side, +/−18°; upper right corner, +/−27°; top, +/−72°; upper left corner, +/−63°) for each frame orientation, giving a total of 9 different possibilities. Alignments within ±4.5° of these values were taken to indicate that the corresponding targeting strategy was being employed and values outside this range indicated random positioning of the line (‘other’). With a random alignment strategy the probability of the alignment falling within any one 9° segment (Fig 1F) would be 0.05, or 5%. The corresponding random probabilities for each of the strategies identified above would therefore be – vertical 5%; sides 10%; corners (right plus left) 20%; top 10%; other 55%.

None of the groups followed a normal distribution and the data were therefore analysed using nonparametric statistical techniques to test for differences between the age groups (Friedman Test – repeated measures) and between genders (Kruskal-Wallis Test) with *post hoc* Dunns Multiple Comparison test. Confidence intervals (95%) for percentages were calculated using the Wilson Method [16].

## Results

### Absolute Errors

**Figure 2:**
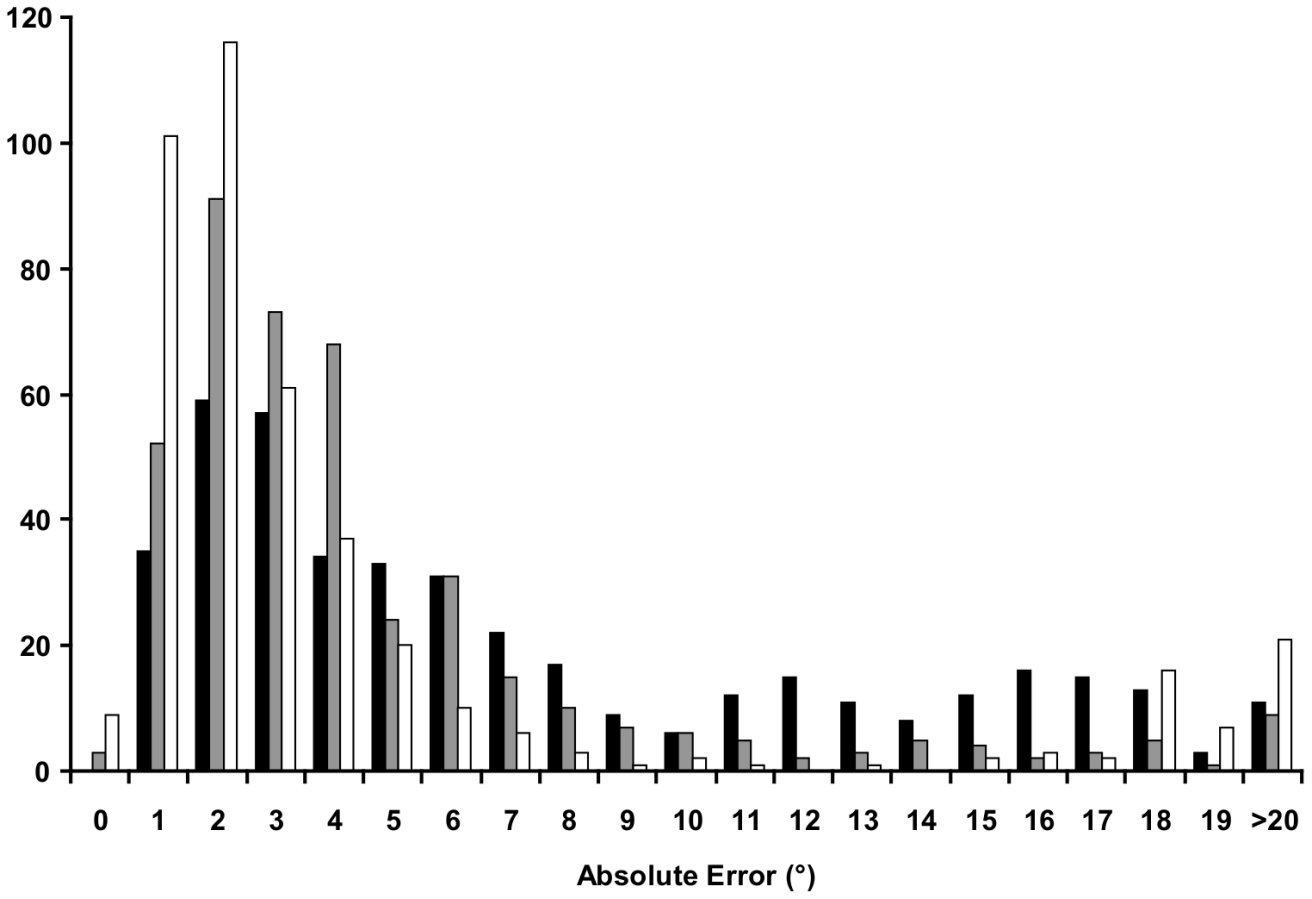
Distribution of mean absolute errors. Grade 2 (black bars), Grade 4 (grey bars), Grade 6 (open bars) - (n = 419 in each Grade).

The mean absolute errors for all 419 subjects showed a wide distribution (Fig 2, Table 1). The distribution of the mean absolute errors showed a similar pattern at all three ages. The mode was 2° and there was a long tail of errors with values greater than 5°. Only when the children reached Grades 4 or 6 did any of the errors fall below 1.0°, and then only in 3 of the Grade 4 children and 9 at Grade 6. At all three ages there were individuals who had mean absolute errors greater than the frame angle of 18°.

**Table 1:** Mean absolute errors recorded for subjects at 2 year intervals.

	Gender	n	Mean (°)	95% CI (°)	Median (°)	Range (°)	Significance
**Grade 2**	Male	214	7.16	6.40 to 7.91	4.8	1.1 to 32.5	-
	Female	205	8.89	7.80 to 9.98	6.4	1.0 to 69.6	-
**Grade 4**	Male	214	4.70	4.16 to 5.24	3.7	0.8 to 29.8	P<0.001
	Female	205	5.77	5.07 to 6.46	4.0	0.9 to 28.9	P<0.001
**Grade 6**	Male	214	5.32	4.20 to 6.44	2.7	0.7 to 85.5	P<0.001
	Female	205	5.80	4.76 to 6.85	3.0	0.8 to 44.7	P<0.001

Significance levels calculated using Friedman Test (Nonparametric Repeated Measures ANOVA), *post hoc* Dunn's Multiple Comparison Test comparing gender groups in Grade 4 and 6 to the corresponding Grade 2 group.

**Figure 3:**
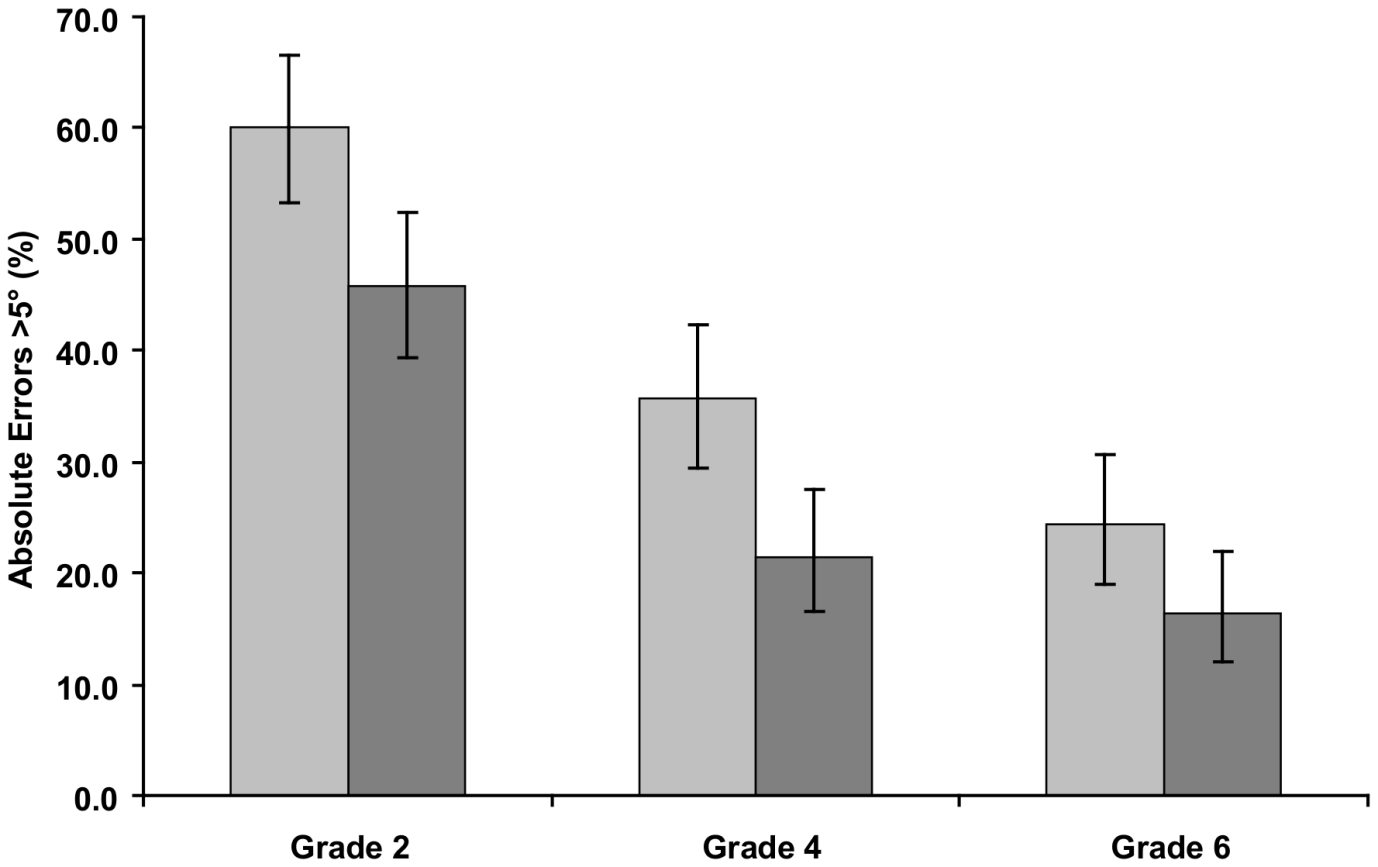
Percentage of subjects having mean absolute errors greater than 5°. Female - light bars; male - dark bars. Error bars show 95% CIs calculated using the Wilson method [16].

The changes in the distribution of errors with age were greatest in those individuals who had large errors in Grade 2. There was a progressive reduction in the percentage of subjects with errors greater than 5° in Grade 4 and Grade 6, compared to Grade 2. Males consistently had smaller absolute alignment errors than females, and at each age had a smaller percentage of errors greater than 5° than the females (Fig 3).

### Individual Changes in Absolute Errors between Grades 2 and 6

**Figure 4:**
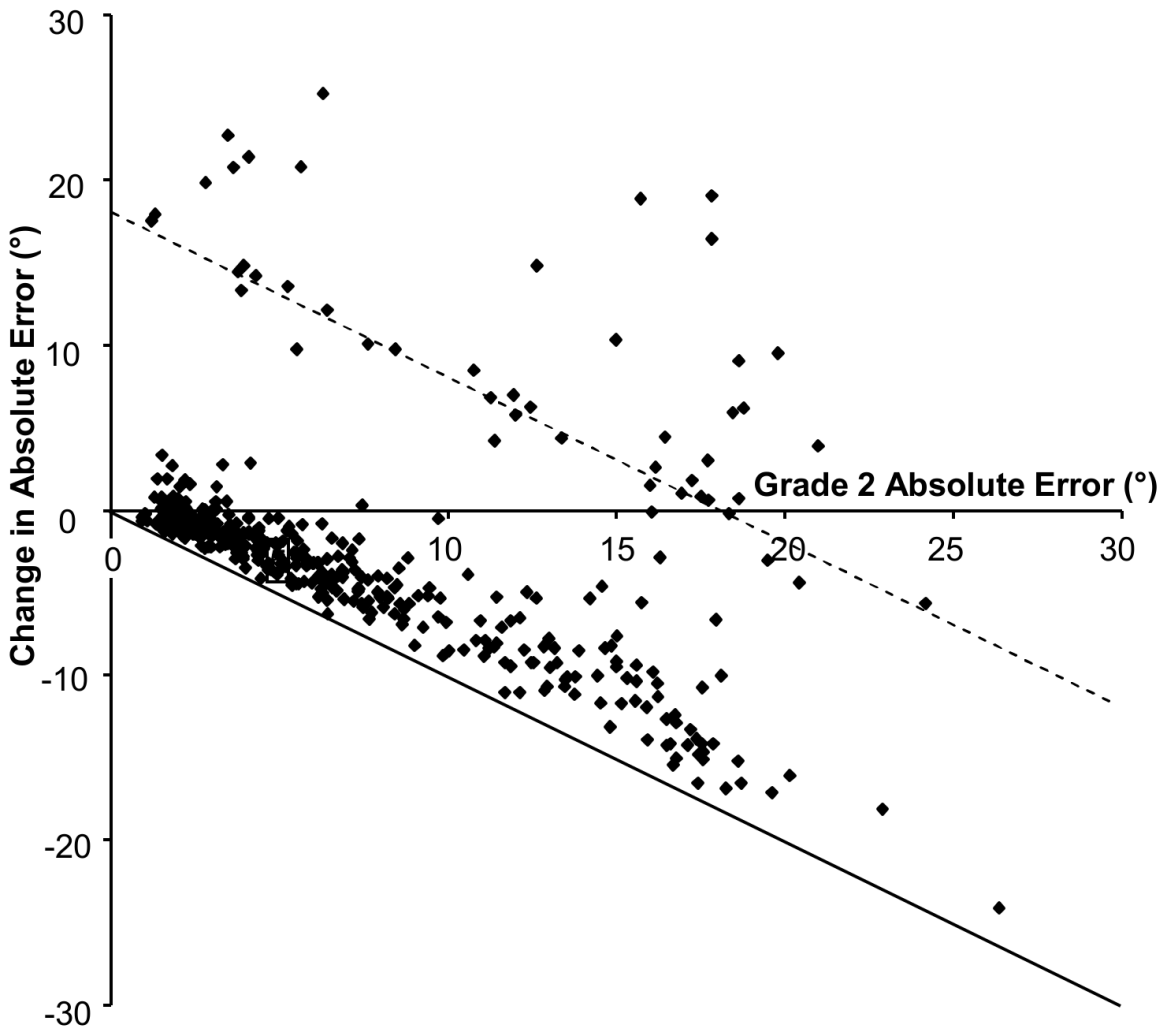
Individual changes in absolute errors. Plot of the change in absolute error between Grade 2 and Grade 6 against the initial Grade 2 error for control subjects. The solid line indicates zero error, the dotted line indicates the frame angle (18°).

To investigate the changes in individual performance over the course of the study the change in each individual's mean absolute error (Grade 6 minus Grade 2) was plotted against their Grade 2 mean absolute error (Fig 4). The graph shows that for most subjects the change in the absolute error between Grade 2 and Grade 6 was negative, reducing their error and bringing it closer to the solid line on the graph which indicates gravitational vertical. This was the case even for many of the individuals who had large errors in the early years (Grade 2). There were however, a number of individuals (82 subjects, 19.6%) whose Grade 6 errors were greater than those at the beginning of the investigation. These are shown as positive values for the change in errors on the graph. Some of these positive values are large (>10°), even in subjects whose initial error was small, and many appear to cluster around a line corresponding to the frame angle (18° - shown as a dotted line in Fig 4). However, use of the absolute errors did not allow it to be determined if the observed changes occurred equally with the frame tilted clockwise or counter clockwise.

### Signed Errors

**Figure 5:**
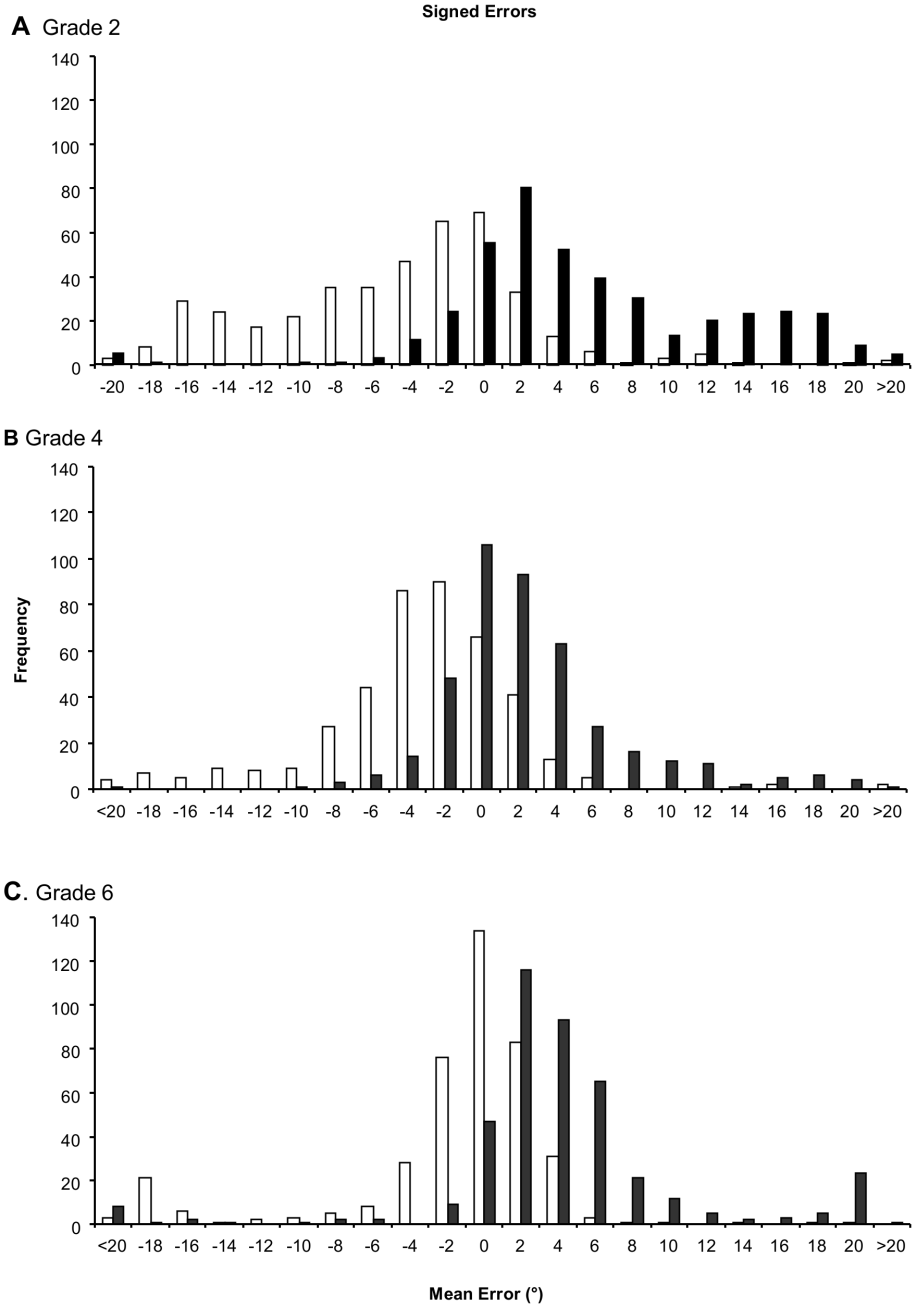
Distribution of signed mean errors. A, Grade 2 (age 7–8 years); B, Grade 4 (age 9–10 years); C, Grade 6 (11–12 years). Open bars – counter clockwise frame tilt; filled bars – clockwise frame tilt. (n = 419 in all cases).

Plots of the distribution of the mean signed errors demonstrated a clear bias of the errors towards the direction of tilt of the frame (Fig 5). The spread of the mean signed errors was broadest in the Grade 2 data, with the Grade 4 and 6 plots displaying progressive condensation towards smaller errors and reductions in the numbers of errors greater than +5° and less than −5°. The Grade 6 data exhibited secondary modes at the angle of frame tilt, +18° and −18°.

### Individual consistency

A measure of the consistency with which an individual aligned the rod was given by the iSD of the errors recorded separately with the frame tilted clockwise and counter clockwise (iSD^+^, iSD^−^), large values indicating poor consistency. Values of iSD greater than 9° (half the frame tilt) were considered to indicate inconsistent positioning and have been excluded from the subsequent analysis. At Grade 2, 39 (9.3%) of the subjects had iSD values >9° when the frame was tilted counter clockwise, and 36 (8.6%) when the frame was tilted clockwise. The corresponding values at Grade 4 were 12 (2.9%) counter clockwise, and 32 (7.6%) clockwise, and at Grade 6, 23 (5.5%) for both counter clockwise and clockwise frame tilts.

**Figure 6:**
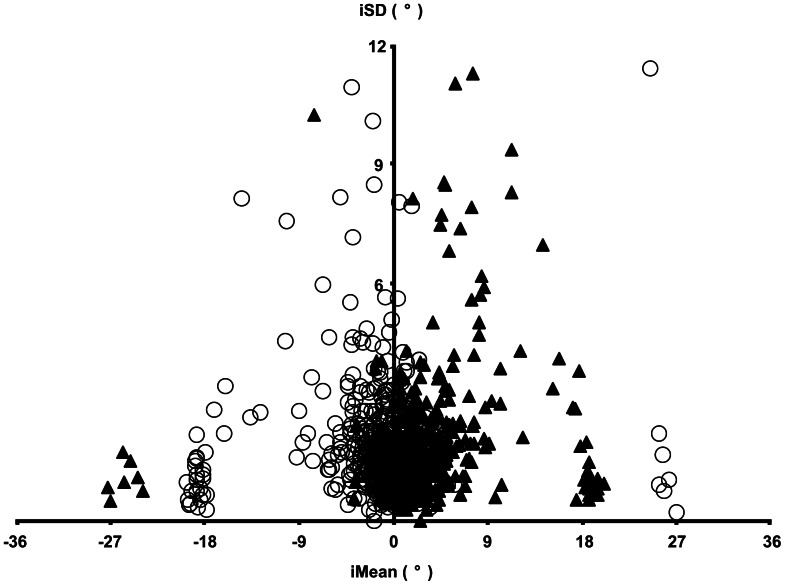
Distribution of individual iMean errors and iSDs in Grade 6. Scatterplot of iMean signed alignment error against iSD, for those subjects having iMean values between −36° and +36°, and iSD values less than 12°. Open symbols frame tilt −18° (n = 403), filled symbols frame tilt +18° (n = 401).

A plot of the iSD against the corresponding iMean for each frame tilt allowed the subjects to be divided into several distinct groups. This was particularly evident at Grade 6 (Fig 6). The majority of subjects had iMean errors within 9° of gravitational vertical (0°), (366 frame counter clockwise, 362 frame clockwise). A second grouping of 26 subjects had iMean values clustered around the angle of the frame tilt (−18° and +18°). Of these 24 had mirror corresponding errors when the frame was tilted in the clockwise and counter clockwise directions.

### Negative visual frame effect

Most of the vertical settings erred in the direction of the frame (Fig 5). However, a number of studies using adults [6],[17],[18],[19] have pointed out the existence of a negative frame effect in which some individuals show errors in the opposite direction to the frame tilt. After exclusion of individuals with inconsistent alignments (iSD >9°) 85 children in the current study showed at least one negative frame effect (iMean) with alignment errors exceeding 3° in the opposite direction to the frame tilt. These have been categorised as mild (3–6°), moderate (6–9°), or strong (>9°). The numbers of children showing negative frame effects was greater in the mild and strong categories than in the moderate category (Table 2), but no pattern of change was discernable between the grades. Of the 111 recordings showing at least one negative frame effect greater than 3° in any grade, 8 individuals showed negative effects with the frame tilted both clockwise and counter clockwise in the same grade. Ten children showed negative frame effects in more than one grade and no child exhibited negative frame effects at all three grades.

**Table 2:** Subjects exhibiting negative frame effects at the three Grades of testing.

			Negative Frame Effect		
	Grade	n	Mild	Moderate	Strong
**Frame −18°**	2	380	8 (2.1)	2 (0.5)	2 (0.5)
	4	407	11 (2.7)	0 (0.0)	2 (0.5)
	6	400	9 (2.3)	0 (0.0)	6 (1.5)
**Frame +18°**	2	383	20 (5.2)	2 (0.5)	4 (1.0)
	4	387	27 (7.0)	5 (1.3)	3 (0.8)
	6	398	3 (0.8)	0 (0.0)	7 (1.8)

The number of subjects (%) showing iMean errors in the opposite direction to the frame tilt. The size of the negative frame effect was categorized as Mild (3°–6°), Moderate (6°–9°) and Strong (>9°). Subjects having iSDs greater than 9° were excluded from the analysis as showing inconsistent responses.

Seven individuals were classified as showing a strong negative frame effect at Grade 6 (Table 3). Of these six had negative errors when the frame was tilted both clockwise and counter clockwise (Fig 6). The magnitude of these negative effects was remarkably consistent between individuals, ranging from 24.5°–27.2° with small iSDs. These individuals had shown few negative frame effects in the preceding two grades. An examination of the errors that would be predicted if the subject aligned the rod with the top right corner of the frame (Fig 1C, 27°) confirmed that this strategy would account for the observed negative frame effects in this group of children.

**Table 3:** Subjects showing strong negative frame effects at Grade 6.

		Grade 2		Grade 4		Grade 6	
Subject	Sex	Frame −	Frame +	Frame −	Frame +	Frame −	Frame +
**HA018**	M	4.6 (4.0)	−0.1 (4.3)	1.9 (2.1)	2.9 (2.1)	**25.3 (0.9)**	**−25.8 (1.7)**
**LA013**	M	−4.4 (4.8)	1.1 (3.2)	−2.1 (1.7)	1.6 (2.0)	**25.8 (0.8)**	**−23.9 (0.8)**
**MG009**	F	−2 (20.0)	**−19.1 (1.7)**	−9.4 (2.0)	−5.0 (10.5)	**25.3 (2.2)**	**−24.4 (1.1)**
**PA013**	F	−18.4 (1.7)	17.6 (1.1)	−7.8 (5.2)	−7.1 (10.7)	−43.9 (23.9)	**−25.7 (0.5)**
**RI013**	M	−5.4 (2.3)	−3.6 (9.6)	−1.9 (2.7)	0.5 (1.5)	**26.9 (0.2)**	**−27.0 (0.5)**
**RI015**	M	8.7 (25.8)	10 (18.1)	−5.1 (4.4)	−6.3 (6.4)	**25.7 (1.7)**	**−25.1 (1.5)**
**TH007**	F	0.4 (5.9)	0.3 (3.6)	−18.3 (1.0)	16.9 (1.0)	**26.2 (1.0)**	**−27.2 (0.9)**

iMean alignment error(°) and (iSD) with the frame tilted counter clockwise (Frame −), and clockwise (Frame +), at the three Grades. Bold  =  large negative frame effects.

### Alignment strategy of individuals with large iSD

At each grade a number of individuals exhibited at least one iSD >9° indicating inconsistent targeting and possibly random alignment of the rod relative to the tilted frame. In Grade 2, forty nine children fell into this category. This number declined to 27 in Grade 4 and 23 in Grade 6 (Table 4). Fifteen children had large iSDs in two of the Grades, and only two had iSDs >9° across all three Grades.

**Table 4:** Frequency of identified alignment strategies used by subjects having iSD >9° at each Grade.

Alignment	Grade 2 (26 F, 23 M)	Grade 4 (18 F, 9 M)	Grade 6 (15 F, 8 M)
Strategy	%	%	%
Vertical	19.4	16.8	16.3
Side	34.4	35.4	37.1
Top	1.8	1.5	8.8
Corners	12.4	9.3	29.6
Other	32.0	36.3	8.3

F – female, M – male

In Grades 2 and 4, 32% and 36.3% respectively of the iSDs in this group were classified in the “Other” alignment strategy, indicating a random element, although this had fallen to 8.3% in Grade 6. Of the frame related targeting strategies, alignment with the sides of the frame was most frequent (Table 4) in all Grades, followed by corners in Grades 2 and 6. The frequency of alignment to vertical was almost constant across the Grades (16.3%–19.4%). Individual subjects were not consistent in the targeting strategy that they employed and all subjects having iSDs >9° used more than one strategy. In all Grades the number of strategies used per subject ranged from 2–4 with a median of 3. Individual subjects who had 50% or more of their iSDs within the ‘Other’ class were considered to be employing a predominantly random approach to rod alignment. In Grades 2 and 4, 26.0% and 33.3% respectively of the children with iSDs >9° were in this group, falling to 4.2% (1 subject) in Grade 6.

### Time

In addition to measuring the errors from vertical, the computer Rod and Frame program also recorded the time taken to align the rod in each presentation. The minimum mean time was 5.5 s (Table 5). This represents the shortest time in which it was possible to rotate the rod from its starting position to vertical. Values below this might suggest that the subject was terminating the session, without trying to align the rod accurately. These data contain no evidence of that occurring. Comparison between the alignment times recorded at the three different ages revealed statistically significant differences between the three sets of data, but the direction of these changes was not consistent.

**Table 5:** Average times per presentation recorded at 2-year intervals.

	Mean (s)	95% CI (s)	Median (s)	Range (s)	Significance
**Grade 2**	12.2	11.7 to 12.6	10.8	5.5 to 336	-
**Grade 4**	10.8	10.5 to 11.2	10.0	5.7 to 25.2	P<0.001
**Grade 6**	11.9	11.6 to 12.2	11.2	5.6 to 25.7	P<0.001

Significance levels calculated using Friedman Test (Nonparametric Repeated Measures ANOVA), *post hoc* Dunn's Multiple Comparison Test comparing Grade 4 to Grade 2, and Grade 6 to Grade 4.

## Discussion

Between the ages of 8 and 12 children are undergoing many developmental changes, they develop better balance and motor coordination, and improved intellectual, mental and social skills. The current study confirmed that there are also changes in the effects of a tilted frame of reference on the estimate of vertical.

### Grouped Data

The report of the absolute Rod and Frame errors in Witkin et al. [3] was confined to a description of the group mean of the absolute deviations. These exhibited large mean errors at age 8, declining until the age of 17 when it levelled off at adult values. The absolute values of the errors in the current investigation are a factor of 2–3 times smaller than those reported by Witkin [3]. This difference has been reported before [18], and is probably the result of the different procedures and angular frame size used between the mechanical and computer Rod and Frame tests. The changes in mean error in the current study followed a similar pattern to that reported by Witkin [3] over the age range 8–12 years. Although recordings were not made at older ages in this study, comparison with data obtained from adults using a similar (but not identical) computer system [20] suggest that there would have been further reductions in the absolute errors over the subsequent 4–5 years. The present study also confirmed that as a group, males consistently had smaller alignment errors than females, although the differences between the genders declined with age, a finding that is similar to those previously reported[3],[4],[5],[9].

### Individual Data

In addition to the large mean and median errors recorded in the current investigation there was also a wide range of values within each grade. One of the problems with working with young children is the question of whether they understand the task of aligning the rod to vertical. Terms such as ‘standing straight up’ [6] or reference to a nearby television tower [5] have been used in place of ‘vertical’ to describe the task. Others have used the analogy of a flagpole [9] or a clown superimposed on the rod [6]. In the current study the task was described as aiming a rocket straight up at the moon.

In the only previous account in which individual children's scores have been considered in detail, Kojima [5] divided the responses into 4 groups: i. ‘relatively independent’ where the child attempted to align the rod to vertical; ii. ‘frame-side dependent’ subjects aligned the rod to one side of the tilted frame; iii. ‘frame-corner dependent’ subjects aligned the rod to an imaginary line constructed between the corners of the tilted frame; iv. ‘others’ for which no clear pattern could be discerned. Similar groupings of individual responses were found in the current study and consideration of the scatter of the values (iSD) around the mean for each individual provided an insight into the targeting strategy used by that child.

The majority of the subjects fell into Kojima's ‘relatively independent’ group [5] with small iMean and iSDs values indicating that they understood the task and were attempting to align the rod to vertical. This group included a number of subjects with mean errors between 5° and 15° who might be considered to fall in the category of ‘frame dependent’, but could equally be argued to have misunderstood the task and to have inaccurately aligned the rod with the surrounding frame.

In a small group of subjects the errors clustered around the frame angle of 18°, (Kojima's ‘frame-side dependent’ group). This might have arisen as a result of misunderstanding or forgetting the task, causing the subject to align the rod to the frame despite being coached during the introductory session to align the rod to vertical. This interpretation receives support from the small iSDs that many of these subjects exhibited, indicating that they were accurately matching the frame angle. A similar clustering of errors around the frame angle has been reported in some elderly subjects [21].

The negative frame effects in which a small number of children consistently aligned the rod 27° in the opposite direction of the frame tilt can be explained as alignment to the frame corners (Kojima's ‘frame-corner dependent’ group'[5]). Kojima's final group – ‘others’ for which no clear pattern could be discerned, corresponds in large part to children in the current study who had large iSD values indicating inconsistent alignments. However analysis of the individual data revealed that in most cases this was not true random alignment of the rod, but the result of switching between different alignment strategies within the recording session, described by Kojima as ‘multiple anchorers’ [5].

### Individual changes with age

By studying individual changes in alignment errors between Grades 2–6 rather than grouped means, it is apparent that in the majority of cases there was a shift towards smaller errors at the older ages, indicating reduced frame dependency. This applied not just to individuals who had small errors at Grade 2, but also many of those who initially had large errors (>10°) at the younger age. This confirms previous reports of increasing Field Independence with age [3],[9], a process which may be associated with the better integration of visual and proprioceptive signals in postural control between the ages of 7 and 10 [22]. However there was also a subgroup of subjects whose errors were larger in Grade 6 than Grade 2. This increase in error was found even in some subjects who had small errors at the younger age. The data points for these individuals tended to be clustered around the line marking the angle of the frame tilt, suggesting that they had become more Frame Dependent at older ages. It is tempting to speculate that these individuals may mature into Frame Dependent adults.

The duration of this study was restricted to 4 years (8–12 years of age). It would be interesting to continue the investigation for a further 5 years to confirm Witkin's findings of a plateau at adult levels in the late teens [3].
